# Anion gap as a prognostic tool for risk stratification in critically ill patients – a systematic review and meta-analysis

**DOI:** 10.1186/s12871-016-0241-y

**Published:** 2016-08-30

**Authors:** Stella Andrea Glasmacher, William Stones

**Affiliations:** 1 School of Medicine, University of St Andrews, Fife, KY16 9TF UK; 2 Malawi College of Medicine, Blantyre, Malawi

## Abstract

**Background:**

Lactate concentration is a robust predictor of mortality but in many low resource settings facilities for its analysis are not available. Anion gap (AG), calculated from clinical chemistry results, is a marker of metabolic acidosis and may be more easily obtained in such settings. In this systematic review and meta-analysis we investigated whether the AG predicts mortality in adult patients admitted to critical care settings.

**Methods:**

We searched Medline, Embase, Web of Science, Scopus, The Cochrane Library and regional electronic databases from inception until May 2016. Studies conducted in any clinical setting that related AG to in-hospital mortality, in-intensive care unit mortality, 31-day mortality or comparable outcome measures were eligible for inclusion. Methodological quality of included studies was assessed using the Quality in Prognostic Studies tool. Descriptive meta-analysis was performed and the I^2^ test was used to quantify heterogeneity. Subgroup analysis was undertaken to identify potential sources of heterogeneity between studies.

**Results:**

Nineteen studies reporting findings in 12,497 patients were included. Overall, quality of studies was poor and most studies were rated as being at moderate or high risk of attrition bias and confounding. There was substantial diversity between studies with regards to clinical setting, age and mortality rates of patient cohorts. High statistical heterogeneity was found in the meta-analyses of area under the ROC curve (I^2^ = 99 %) and mean difference (I^2^ = 97 %) for the observed AG. Three studies reported good discriminatory power of the AG to predict mortality and were responsible for a large proportion of statistical heterogeneity. The remaining 16 studies reported poor to moderate ability of the AG to predict mortality. Subgroup analysis suggested that intravenous fluids affect the ability of the AG to predict mortality.

**Conclusion:**

Based on the limited quality of available evidence, a single AG measurement cannot be recommended for risk stratification in critically ill patients. The probable influence of intravenous fluids on AG levels renders the AG an impractical tool in clinical practice. Future research should focus on increasing the availability of lactate monitoring in low resource settings.

**PROSPERO registration number:**

CRD42015015249. Registered on 4th February 2015.

**Electronic supplementary material:**

The online version of this article (doi:10.1186/s12871-016-0241-y) contains supplementary material, which is available to authorized users.

## Background

Much research has focussed on the prognostic value of serum lactate estimation in critically ill patients [1]; however, in the context of work in low resource settings we have noted that facilities for lactate and blood gas analysis are frequently not available, prompting a search for alternative risk stratification tools. The anion gap (AG) is an easily calculated marker of metabolic acidosis based on analytes typically available from routine chemistry analysis. It may have potential as a risk stratification tool to identify sick patients at risk of deterioration, who would benefit from further management whilst pathophysiological processes are still reversible. The AG reflects the concentration of unmeasured anions as calculated by the formula Na^+^ - (Cl^−^ + HCO_3_^−^). Inclusion of potassium in the formula is recommended where its concentration is abnormally high or low [2]. In healthy subjects, the unmeasured anions or “gap” is mostly made up of albumin; however, hypoalbuminaemia, commonly observed in critically ill patients, can lower the AG and mask an acidosis. Feldman and colleagues therefore recommended that the AG should be corrected for albumin [3].

In metabolic acidosis, addition of fixed acids leads to a rise of the AG: while the proton within the acid combines with bicarbonate, the conjugate base contributes to the unmeasured anions. Metabolic acidosis is common in critically ill patients and is a strong predictor of prognosis [4]. Maciel and Park observed that unmeasured anions accounted for the majority of metabolic acidosis in both intensive care unit (ICU) survivors and non-survivors, whereas lactate accounted for only a quarter of acidosis [5]. AG may thus have potential as a risk stratification tool, especially if corrected for albumin.

The validity of the AG as a predictor of mortality has been studied and has been compared to other indices of acid–base balance, especially Stewart’s strong ion gap [6]. However, the strong ion gap is more cumbersome and expensive to measure than the AG and is thus less suitable as a risk stratification tool in low resource settings. In studies with contrasting findings, AG was noted to be a very strong predictor of mortality [7] or of limited value with neither the AG nor the strong ion gap effective as predictors of in-hospital mortality [8]. Furthermore, it has been noted that studies conducted in countries where gelatin-based intravenous fluids are routinely used, such as the UK and Australia, failed to show an association between the strong ion gap and mortality whereas studies conducted in settings where such fluids are not routinely used, especially the USA were able to demonstrate an association [9, 10]. Gelatins are an exogenous source of unmeasured anions [11] and an increase in AG after gelatin infusion has been demonstrated in animal experimental studies [12].

Recently, a large study of 18,985 patients found that ∆AG, defined as the difference in AG between pre-hospital admission and critical care admission, was a robust predictor of all-cause mortality, where the pre-hospital AG was determined between seven and 365 days before admission [13]. However, this approach requires adequate documentation and a laboratory database, which are unlikely to be available in resource-limited settings.

In the present systematic review and meta-analysis we therefore aimed to determine the validity of a single AG measurement as a risk stratification tool predicting 31-day mortality in-hospital mortality, in-ICU mortality and comparable outcome measures in adult patients admitted to critical care settings. We also aimed to compare the prognostic validity of the observed and albumin-corrected AG. Although the AG as a risk stratification tool would be mainly applicable to low income countries, this systematic review and meta-analysis does not limit itself to studies conducted in such countries as the main focus lies on the scientific validity of the AG as a risk stratification tool.

## Methods

### Protocol registration

This systematic review and meta-analysis adheres to the “preferred reporting items for systematic reviews and meta-analyses” (PRISMA) standards [14]. A protocol was registered with PROSPERO, registration number CRD42015015249.

### Search strategy, study selection and data extraction

We searched the electronic databases of Medline, Embase, Scopus, Medion, The Cochrane Library, Web of Science and regional bibliographic databases including African Index Medicus, Latin America and the Caribbean (LILACS), IndMed, Index Medicus for South East Asia Region (IMSEAR) and Western Pacific Region Index Medicus (WPRIM). In addition, journals specialising in the fields of critical care, anaesthetics, emergency medicine and intensive care medicine were searched electronically. Searches were performed for studies that were conducted on humans and published in English, German or French using the search terms “anion gap”, “unmeasured anions” and “unidentified acids”. The initial search was performed in January 2015 and the search was subsequently updated in May 2016. All search results were initially screened by abstract and title and those considered relevant subsequently underwent full-text screening. To identify further relevant studies, reference lists were reviewed, citation searches were performed and citation alerts were set up for all articles considered relevant after full-text screening.

Studies, conducted in any acute care clinical setting, were eligible for inclusion if they were published within the last 15 years, reported measurement of the observed and/or corrected serum AG in adult patients and mortality defined as “in-hospital mortality”, “in ICU mortality” or, if a time-frame was stated, death within up to 31-days of hospital admission. The latter definition was chosen where both outcomes were reported within a single study. Case studies, case–control studies and studies whose main focus was a hyperglycaemic emergency, poisoning or renal failure were excluded.

Data extraction was performed by a single reviewer, SG. A second reviewer, WS, independently extracted data from 10 % of the studies selected using a random number generator. Corresponding authors were contacted where necessary to discuss missing or unclear data.

### Assessment of methodological quality and risk of bias

Two reviewers (SG and WS) independently graded the methodological quality and risk of bias of included studies using a modified version of the Quality In Prognostic Studies (QUIPS) tool [15]. This tool assesses the risk of bias of prognostic studies in six domains: study participation, study attrition, prognostic factor measurement, outcome measurement, confounding, and statistical analysis and reporting. Each study was rated as being at high, moderate or low risk of bias in each domain. Disagreements were resolved by discussion between the two reviewers.

### Statistical analysis

Area under the ROC curve (AUCs), odds ratios (ORs) and mean differences were pooled in random or fixed effects generic inverse variance models for the observed and corrected AG. The I^2^ test was used to quantify heterogeneity. A fixed-effects model was used where the I^2^ was below 30 %; otherwise, a random-effects model was used. Meta-analysis of ORs and mean differences was undertaken in Review Manager version 5.3 (The Cochrane Collaboration, 2014, Copenhagen), while AUCs were pooled in StatsDirect version 2.8.0 (England: StatsDirect Ltd. 2013); an AUC of ≥0.8 was considered to denote good discriminatory power. Pooled estimates were not presented in forest plots due to high heterogeneity in the meta-analyses of AUC and mean difference; in the results section the pooled estimates are reported together with their respective 95 % confidence intervals (CIs). Subgroup analysis was undertaken to assess whether heterogeneity between studies could be explained by the following study characteristics: patient age, study setting, quantity of intravenous fluids received, determination of the AG before the initiation of hospital-based treatment, the routine use of gelatin-based intravenous fluids in the study country, choice of outcome measure, publication date and overall mortality.

Statistical significance testing for subgroup differences employed the unpaired *t*-test in GraphPad® QuickCalc Web Calculator (La Jolla California USA) [16]. Probabilities were two-tailed and a probability of less than 0.05 was considered statistically significant. No adjustments were made for multiple comparisons. Sensitivity analysis was undertaken to assess the effect of including retrospective studies and studies at high risk of attrition bias in the meta-analysis. Funnel plots were visually inspected for evidence of publication bias.

## Results

### Study selection and characteristics of included studies

**Fig. 1:**
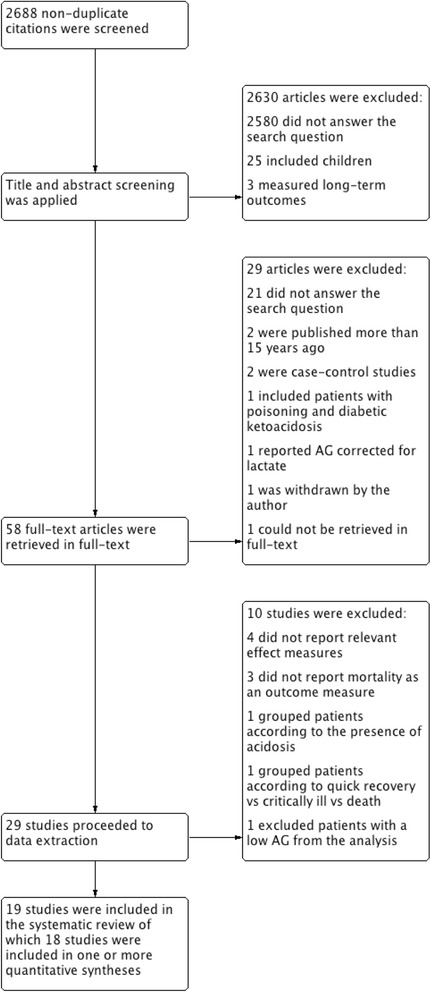
Flow chart summarising the search and study selection process. DKA = diabetic ketoacidosis; SOFA = sequential organ failure assessment score

The study selection process is summarised in Fig. 1. In total, the search yielded 2688 non-duplicate publications; 2630 articles were excluded after title and abstract screening thus 58 articles were retrieved in full-text. Twenty-nine articles were excluded during full-text screening, leaving 29 studies that were subjected to data extraction. Ten studies were excluded during data extraction and thus 19 studies were included in the systematic review, of which 18 were included in one or more quantitative syntheses.

Table 1 illustrates the characteristics of included studies. A majority of studies were conducted in high income countries [7, 8, 16–26] while three studies were conducted in middle income countries [27–29] and one in a low income country [30]. Studies were conducted in the following settings: ICU (10 studies), trauma centre (5 studies), coronary care unit/intensive cardiac care unit (3 studies) and Accident and Emergency department (1 study). Five studies accounted for the effect of intravenous fluids on AG levels: in one study no patient received more than 400 ml of any intravenous fluid before the AG was measured [7], in two studies patients receiving more than 250 ml or 500 ml of intravenous fluids respectively were excluded from the analysis [16, 30]. Two studies stated that the AG was determined before hospital based management, including intravenous fluids, was initiated [21, 29]. The remaining studies did not report on the quantity of intravenous fluids received by their study cohorts. No study reported both in-hospital and a time-frame specific mortality. One study [29] failed to define the outcome measure, reporting it as “mortality”. Sensitivity analysis was carried out to determine whether inclusion of this study affected the pooled effect measure. The risks of bias ratings are displayed in Table 2. Risk of attrition bias and confounding were the most poorly rated domains. There were no disagreements between review authors on data extraction.

**Table 1:** Characteristics of included studies

First author/year	Nr	Country	Setting and most frequent reasons for admission	Study design	Age (mean or mdn)	*Sample size*	Male (%)	Outcome (mortality)	Total mortality (%)	Severity of illness (mean score ± SD or mdn and range or IQR)
Antonini 2008 [17]	1	Italy	General ICU admissions: 36 % trauma; 26 % cerebrovascular disease; 14 % sepsis	Pro	Mean: 53	136	71	28-day	27	SOFA: 6 (range 0–18) SAPS II: 40 (range 6–76)
Attanà 2013 [18]	2	Italy	STEMI patients with persistent cardiogenic shock after primary PCI admitted to ICCU	Pro	Mean: 73	63	62	In-ICCU	49	APACHE II: 20.6 ± 12.4
Boniatti 2011 [27]	3	Brazil	General ICU admissions: 64 % medical admissions; 27 % sepsis; 24 % elective surgery; 12 % emergency surgery	Pro	Mean: 56	175	53	In-hospital	37	APACHE II: 20.8 ± 8.0 SOFA score: 6.2 ± 3.8
Cusack 2002 [19]	4	UK	General ICU admissions: 17 % respiratory failure; 11 % post-cardiac arrest; 8 % trauma	Pro	Mean: 61	100	NA	28-day	31	APACHE II: 20.5
Dondorp 2004 [29]	5	Vietnam	Patients with severe falciparum malaria admitted to ICU	Pro	Mdn: 31	268	80	Not defined	17	GCS < 11: 51 % 8 % Haemodynamic shock^a^
Dubin 2007 [37]	6	Argentina	General ICU admissions: 56 % medical admissions; 35 % elective surgery; 9 % emergency surgery	Pro	Mean: 65	935	49	30-day	11	APACHE II: 13 ± 7 SOFA: 3 ± 3
FitzSullivan 2005 [20]	7	USA	Trauma ICU admissions: 60 % blunt trauma	Retro	Mean: 36	3102	81	In-hospital	17	APACHE II: 26.1 ± 10.5 ISS: 20.4 ± 12.9
Hucker 2005 [21]	8	UK	A&E admissions: 46 % medical admissions; 17 % elderly care; 16 % discharged	Pro	Mean: 67	672	NA	In-hospital	12	93 % alert on AVPU scale
Kaplan 2004 [7]	9	USA	Trauma patients requiring vascular repair of torso or extremities, trauma centre: 83 % penetrating trauma	Retro	Mean: 32	282	NA	28-day	23	ISS: 15.8 ± 11.0
Kaplan 2008 [16]	10	USA	Major trauma patients, trauma centre: 59 % blunt trauma	Retro	Mean: 33	78	44	28-day in hospital	33	ISS: 8.9 ± 7.3
Lazzeri 2010 [22]	11	Italy	STEMI patients admitted to ICCU at tertiary centre undergoing primary PCI	Pro	Mdn: 67	445	75	In-hospital	10	92 % Killip class I-II 8 % Killip class II-IV 41 % complications in ICCU
Lipnick 2013 [13]	12	USA	General ICU admissions: 57 % medical; 44 % surgical; 16 % sepsis	Retro	Mean: 65	664	55	30-day	15	33 % no organ failure 53 % 1–2 organs failed 14 %% ≥ 3 organs failed^b^
Martin 2013 [23]	13	Germany	Surgical ICU admissions: 17 % maxillofacial surgery; 13 % ENT; 12 % neurosurgery	Retro	Mean: 59	1551	54	In-hospital	9	Average length of stay in ICU: 4.2 days
Martin 2005a [25]	14	USA	Surgical ICU admissions: 56 % abdominal; 18 % vascular; 10 % thoracic	Retro	Mean: 52	2291	61	In-ICU	8	APACHE II: 21.8 ± 9.7 SAPS: 16.8 ± 8.8
Martin 2005b [24]	15	USA	Trauma patients, trauma centre: 65 % blunt trauma	Retro	Mean: 38	427	79	In-hospital	10	ISS: 23 ± 23
Novovic 2014 [28]	16	Serbia	ICU patients requiring mechanical ventilation	Retro	Mean: 60	142	47	28-day	52	APACHE II: 16.2 ± 6.4
Rocktaeschel 2003 [8]	17	Australia	General ICU admissions: 91 % respiratory; 54 % gastrointestinal; 51 % cardiovascular	Retro	Mdn: 65	300	58	In-hospital	28	APACHE II: 17 (IQR 14 – 22)
Sahu 2006 [26]	18	USA	Patients with acute MI admitted to coronary care unit: 65 % STEMI	Retro	Mean: 63	773	62	In-hospital	11	5 % cardiogenic shock
Shane 2014 [30]	19	Uganda	Major trauma patients, trauma centre: 65 % road traffic accidents; 35 % assault	Pro	Mean: 26	93	81	In-hospital	34	ISS: 25.4 ± 8.3

*APACHE II* Acute Physiology and Chronic Health Evaluation, *AVPU* alert, verbal, pain, unresponsive, *ENT* ear, nose and throat, *GCS* Glasgow coma scale, *ICCU* intensive cardiac care unit, *ICU* intensive care unit, *IQR* interquartile range, *ISS* Injury Severity Score, *Mdn* median, *MI* myocardial infarction, *PCI* percutaneous coronary intervention, *Pro* prospective, *Retro* retrospective, *SAPS* simplified acute physiology score, *SD* standard deviation, *SOFA* sequential organ failure assessment, *STEMI* ST-elevation myocardial infarction

^a^Based on data of previously published original study including 346 patients [38]

^b^Based on data from entire study cohort of 18,995 patients

**Table 2:**
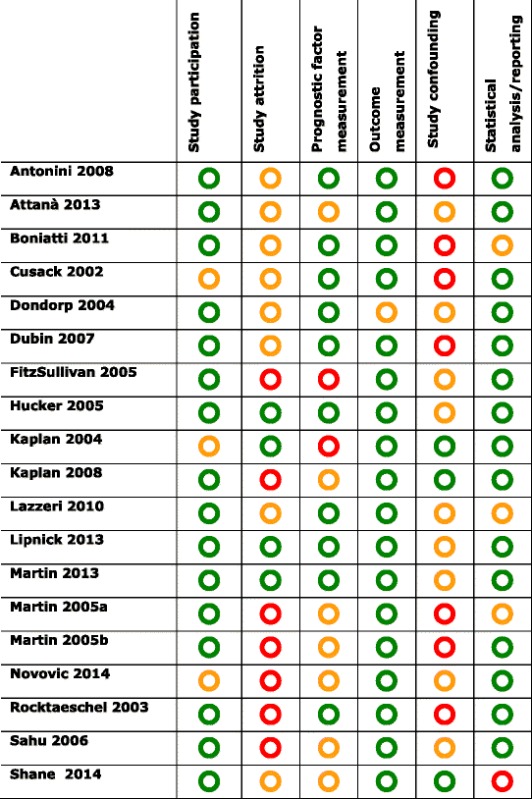
Risk of bias rating

Green, Yellow and Red refer to low, moderate and high risk of bias respectively

### Prognostic ability of the AG to predict mortality

Owing to the high heterogeneity identified in the meta-analyses of AUC and mean difference, the pooled effect measures reported in this section should not be interpreted. Overall, three studies reported good discriminatory ability of the AG to predict mortality, of which two studies were included in meta-analysis [7, 16] and one study allowed the calculation of an OR for a specific AG threshold [22]. The former two studies were responsible for a large proportion of the statistical heterogeneity; both studies were conducted in young patients in the same trauma centre and only patients receiving less than a specified volume of intravenous fluids were included in the analysis. The latter study was conducted in patients with ST-elevation myocardial infarction undergoing percutaneous coronary intervention. The remaining 16 studies reported poor to moderate ability of the AG to predict mortality.

**Fig. 2:**
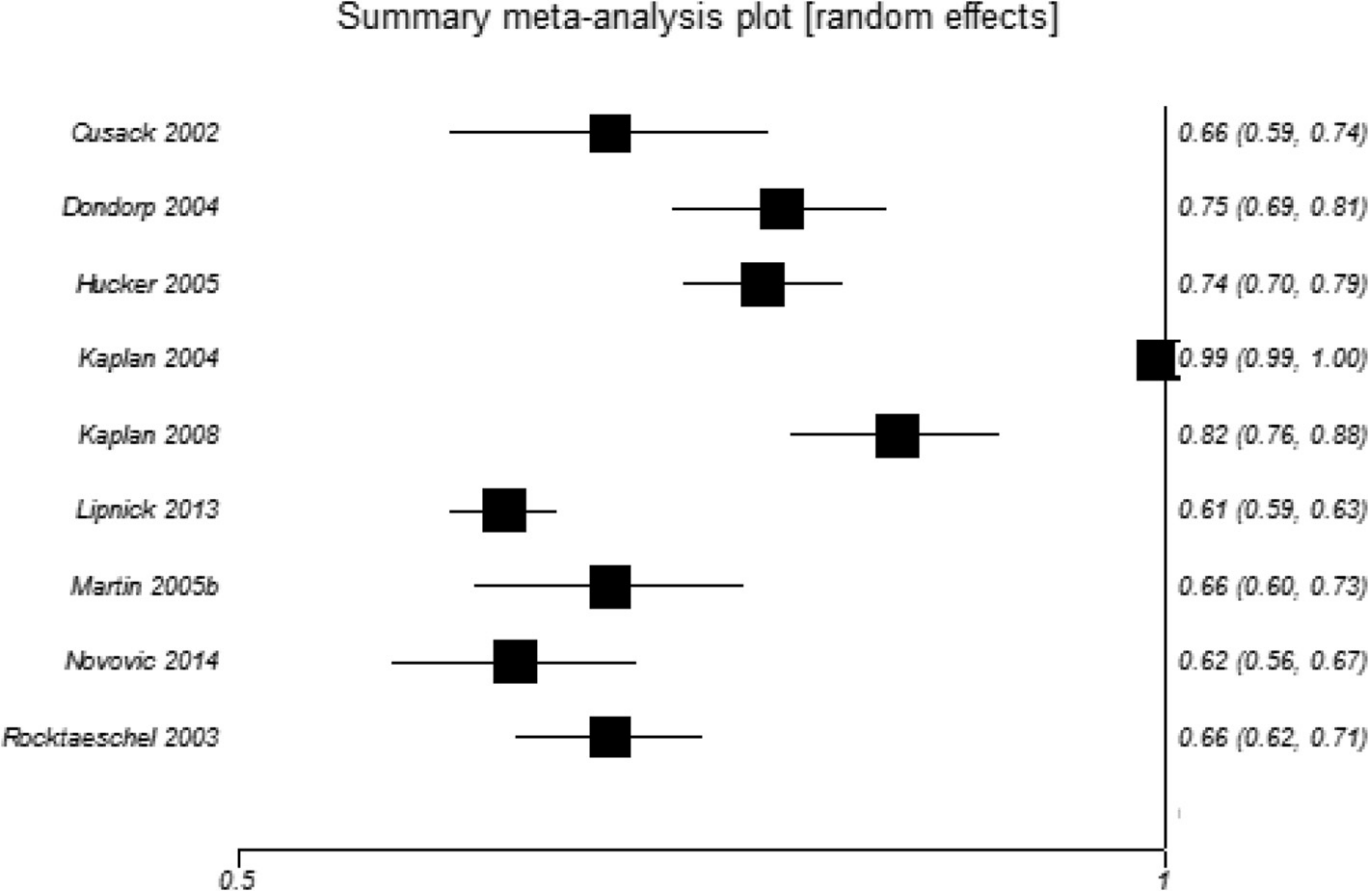
Forest plot of area under the ROC curves (AUCs) for observed AG predicting mortality. Forest plot of a random effects meta-analysis of AUCs for the observed AG predicting mortality; I^2^ = 99 %. In view of the high heterogeneity a pooled effect estimate is not shown

Nine studies reported AUCs for the observed AG (Fig. 2). Meta-analysis yielded a summary AUC of 0.72 (95 % CI 0.59 to 0.86). Heterogeneity was very high (I^2^ = 99 %) but reduced to I^2^ = 68 % when the two studies by Kaplan and Kellum were excluded from the analysis [7, 16]. Six studies reported AUCs for the corrected AG (Additional file 1: Figure S1). The summary AUC was estimated as 0.67 (95 % CI 0.62 to 0.71) and heterogeneity was high (I^2^ = 67 %).

**Fig. 3:**
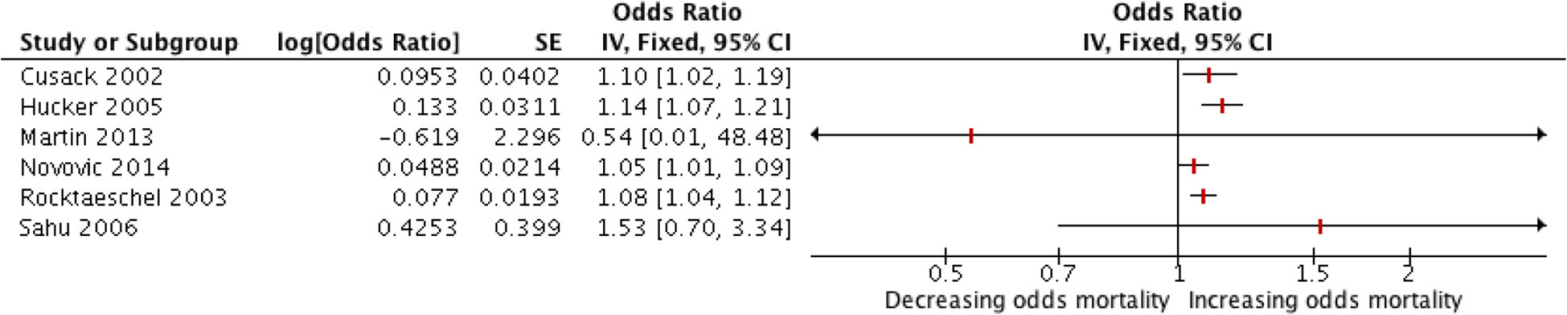
Forest plot of odds ratios (ORs) for observed AG predicting mortality. Forest plot of a fixed effects meta-analysis of ORs derived by univariate logistic regression for the observed AG predicting mortality; I^2^ = 0 %. In view of the high heterogeneity in meta-analyses of other effect measures a pooled effect estimate is not shown

Six studies reported ORs derived by logistic regression modelling for the observed AG (Fig. 3); five reported univariate logistic regression ORs whilst one study reported an OR adjusted for age. The summary OR was 1.08 (95 % CI 1.06 to 1.11); results were homogenous (I^2^ = 0 %) but it should be noted that the two studies by Kaplan and Kellum were not included in this analysis as neither study reported the OR. Four studies reported ORs derived by univariate logistic regression for the corrected AG (Additional file 2: Figure S2). The summary OR was 1.10 (95 % CI 1.07 to 1.13) and heterogeneity was very low (I^2^ = 5 %). Data reported in the study of Lazzeri and colleagues allowed the calculation of an OR for a specified AG positivity threshold. This yielded an OR of 2.8 (95 % CI 1.5 to 5.5) for an AG positivity threshold of 11 mEq/L [22].

**Fig. 4:**
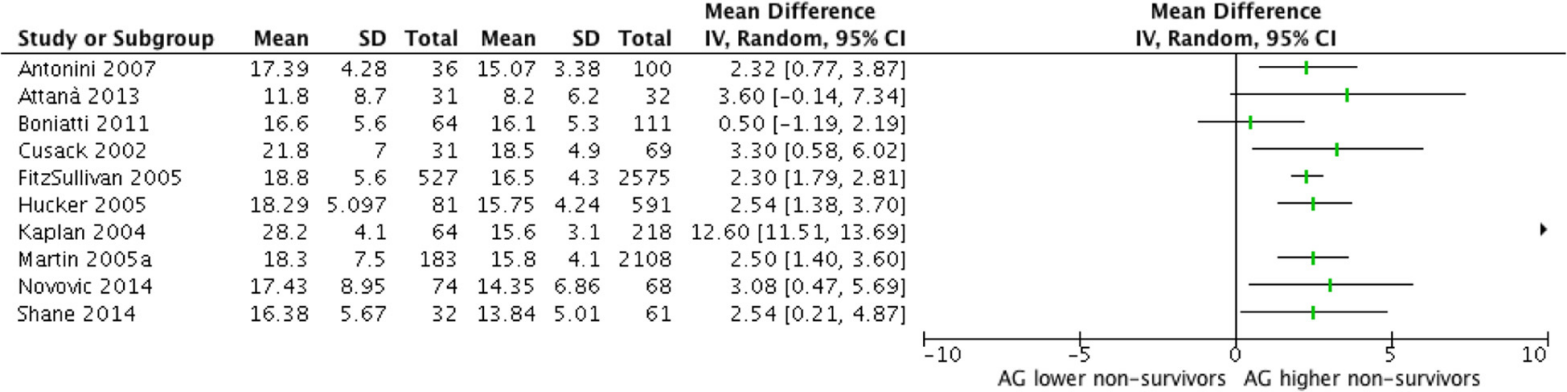
Forest plot of mean differences for observed AG predicting mortality. Forest plot of mean differences in observed AG between survivors and non-survivors; I^2^ = 96 %. In view of the high heterogeneity a pooled effect estimate is not shown

Mean difference was the most frequently reported effect measure with ten studies reporting it for the observed AG (Fig. 4). The summary mean difference was 3.55 mEq/L (95 % CI 1.08 to 6.02). Heterogeneity was high (I^2^ = 97 %); however, excluding the study by Kaplan and Kellum [7] completely eliminated heterogeneity (I^2^ = 0 %). The mean difference for corrected AG was reported by three studies (Additional file 3: Figure S3) and the summary mean difference was estimated as 3.25 mEq/L (95 % CI 1.53 to 4.96) with homogeneous results (I^2^ = 0 %).

Sensitivity analysis showed that including retrospective studies and studies at risk of attrition bias did not affect the summary AUC. Including prospective studies only (six studies) yielded a summary AUC of 0.73 (95 % CI 0.69 to 0.78). Similarly, excluding studies rated at high risk of attrition bias (four studies) yielded a summary AUC of 0.75 (95 % CI 0.54 to 0.96). Excluding the study by Dondorp and colleagues [31] with an undefined outcome measure (“mortality”) yielded an AUC of 0.72 (95 % CI 0.56 to 0.88).

AUC of observed AG was chosen for subgroup analysis; results are shown in Table 3. The quantity of intravenous fluids given to a patient had the strongest influence on the summary AUC. Studies excluding patients who received more than a specified volume of intravenous fluids [7, 16] reported a significantly higher summary AUC than studies not excluding patients for this reason (*P =* 0.0008), but heterogeneity remained high in both subgroups. For the subsequent analysis, studies restricting intravenous fluids [7, 16] and studies measuring the AG before initiation of hospital-based management [21, 29] were combined in a subgroup and compared to studies that did not account in any way for the effect of intravenous fluids on the AG. The former subgroup yielded a significantly higher summary AUC than the latter subgroup (*P <*0.0001). Heterogeneity remained high in the former subgroup (I^2^ = 98 %), though results in the latter subgroup were homogenous (I^2^ = 0 %). The summary AUC of studies conducted in countries where gelatin-based resuscitation fluids are routinely used [8, 19, 28] is not significantly different to that of studies conducted in countries where gelatins are not routinely used [13, 24] (*P =* 0.33). Studies in which intravenous fluids were restricted or in which the AG was measured before initiation of hospital-based treatment were not included in the latter comparison. The observed AG appears to be a slightly better predictor of mortality among younger patients (*P =* 0.011) and those admitted to trauma centre settings (*P =* 0.0235). Subgroup analysis showed no significant difference between studies reporting in-hospital mortality and those reporting a time-framed mortality (*P =* 0.65); similarly, no significant difference was found between studies in which overall mortality was below 30 % and above 30 % (*P =* 0.89) or between studies published before and after the year 2005 (*P =* 0.43); and heterogeneity remained high in all subgroups. Funnel plots did not show evidence of publication bias (Additional file 4: Figure S4 and Additional file 5: Figure S5).

**Table 3:** Results of subgroup analysis

Study characteristic	Groups	Studies (nr)	Total sample size	Pooled AUC (95 % CIs)	I^2^ test	*P*-value
Study setting	Trauma patients	9, 10, 15	787	0.83 (0.68, 0.99)	97 %	0.0235
	ICU patients	4, 5, 12, 16, 17	1474	0.66 (0.59, 0.73)	69 %	
Age	Mean/Median age 30–40 years	5, 9, 10, 15	1055	0.81 (0.69, 0.93)	97 %	0.0114
	Mean/Median age 60–70 years	4, 8, 12, 16, 17	1878	0.66 (0.60, 0.71)	70 %	
Intravenous fluids restriction	Restriction	9, 10	356	0.91 (0.8, 1.0)	95 %	0.0008
	No restriction	4, 5, 8, 12, 15, 16, 17	2573	0.67 (0.62, 0.72)	68 %	
Intravenous fluids restriction and AG measured before treatment initiation	Restriction and AG measurement before hospital treatment initiation	5, 8, 9, 10	1296	0.83 (0.73, 0.93)	98 %	<0.0001
	No restriction or AG measured after treatment was commenced	4, 12, 15, 16, 17	1633	0.63 (0.60, 0.66)	0 %	
Routine use of gelatin-based intravenous fluids in study country	Gelatins not routinely used	12, 15	1091	0.62 (0.58, 0.65)	0 %	0.3344
	Gelatins routinely used	4, 16, 17	542	0.65 (0.6, 0.7)	0 %	
Outcome measure	Time frame stated e.g. 31-day or 28-day mortality	4, 9, 10, 12, 16	1266	0.74 (0.52, 0.96)	99 %	0.6518
	In-hospital mortality	8, 15, 17	1399	0.69 (0.64, 0.75)	44 %	
Overall mortality in study population	Below 30 %	5, 8, 9, 12, 15, 17	2613	0.74 (0.55, 0.93)	99 %	0.8856
	Above 30 %	4, 10, 16	320	0.70 (0.57, 0.84)	83 %	
Date of publication	Before and including 2005	4, 5, 8, 9, 15	1710	0.76 (0.59, 0.94)	98 %	0.4325
	2006 and after	10, 12, 16, 17	1184	0.67 (0.58, 0.77)	87 %	

*CIs* confidence intervals, *ICU* intensive care unit

## Discussion

This systematic review and meta-analysis does not support the use of a single AG measurement for risk stratification in critically ill patients. Quantitative synthesis was limited by significant statistical heterogeneity, which, following a series of subgroup analyses, could be partially explained by the quantity of intravenous fluids received by study patients. Studies differed substantially with regards to setting, presumed use of gelatin-based intravenous fluids as well as the age and mortality rate of their patient cohorts; however, in our analysis none of these factors fully accounted for the high degree of heterogeneity. Disease severity was not consistently characterised across studies and could therefore not be analysed in subgroup analysis. Overall, the high degree of unexplained heterogeneity, poor quality of primary studies and poor to moderate discriminatory power of the AG reported by the majority of studies suggest that there is insufficient evidence to recommend the use of the AG in clinical practice. Owing to the small number of studies that calculated a corrected AG, we were unable to determine whether correction of the AG for albumin improves its predictive ability.

In subgroup analysis, a highly statistically significant difference was seen between studies accounting for intravenous fluids by means of fluid restriction or by measuring the AG before the initiation of hospital-based management and studies that did not account for quantity of intravenous fluids by any means. This indicates that intravenous fluids may have blurred the association between AG and mortality. Administration of normal saline lowers the AG because addition of NaCl to the plasma increases the baseline chloride concentration proportionately more than the baseline sodium concentration, owing to the differences in volume of distribution between the two ions [4]. This effect may not be seen with more balanced fluids such as Hartman’s solution but, given that normal saline is commonly used in clinical practice, a risk stratification tool that is considerably affected by saline infusion is impractical. However, the validity of this subgroup analysis is limited by its observational nature and relatively small number of studies contained in each subgroup. Therefore, other confounders may have accounted for these results. Furthermore, the study by Shane and colleagues [30] also employed intravenous fluid restriction but found no predictive effect of the AG; however, only mean difference in AG between survivors and non-survivors but not AUC was reported. Further research would be required to determine more conclusively the extent to which the AG is affected by intravenous fluids.

Other than intravenous fluid restriction, our subgroup analysis did not identify factors accounting for the high statistical heterogeneity in the meta-analysis of AUC. A small effect of study setting and mean/median age on the pooled AUC was observed; however, the associated probabilities were >0.01 where no adjustments were made for multiple comparisons and in both analyses one subgroup contained the two studies by Kaplan and Kellum [7, 16]. Therefore, we consider the observed differences most likely to have arisen due to chance or confounding. Notably, no significant effect of outcome measure or publication date was observed on the pooled AUC. This supports the appropriateness of including studies reporting a time-framed mortality and in-hospital mortality and studies published at different times over the past 15 years. As parameters denoting disease severity were not consistently reported across studies, we divided studies according to overall mortality in subgroup analysis. No difference in pooled AUC was seen between studies reporting mortality rates above and below 30 %; however, overall mortality is a suboptimal indicator of severity of illness. Therefore, the contribution of disease severity to the observed statistical heterogeneity remains unclear.

Another important factor limiting the ability of the AG to predict clinical outcomes is its considerable baseline variability amongst healthy people. To address this, Kraut and Nagami suggested comparing the AG during an acute admission to the “personal AG” measured when the individual was in good health [2]. Dynamic AG indices, describing not only the magnitude of acid–base disturbance but also trends over time, are better predictors of mortality, as shown by the large study by Lipnick and colleagues including 18,995 patients [13]. For a subset of patients in this study (*n =* 664), the predictive ability of a single AG measurement was also reported and was shown to be poor (AUC 0.61). However, implementation of the systems required to support a “personal AG” would be challenging in low resource settings.

A rise in the AG in critically ill patients was long thought to be predominantly due to lactic acidosis, yet several studies reported poor sensitivity of the AG in detecting hyperlactataemia defined by a lactate threshold of 2.5 mmol/l [31–34]. The AG was an excellent predictor of severe hyperlactataemia defined as lactate above 4 mmol/l [32] or 5 mmol/l [8]; however, Nichol and colleagues found that a higher lactate concentration even within a normal reference range of 2 mmol/l independently predicts mortality [35]. The AG may thus miss patients at risk of mortality, as a considerable degree of hyperlactataemia is required to push the AG outside its normal reference range if the baseline AG is low [2]. This is in keeping with the extreme difference in lactate levels between survivors and non-survivors observed in the study reporting the highest predictive value of AG [7]. Other studies reported smaller, albeit mostly statistically significant, differences in lactate levels between survivors and non-survivors.

### Limitations

The methodological quality of primary studies was generally poor. Most studies were rated at moderate or high risk of attrition bias and sampling bias as a result of failure to quantify missing outcome or prognostic data, especially in retrospective studies, where case notes with missing information are less likely to have been available. This may have affected our results where information was missing in a non-random manner. Similarly, risk of confounding was moderate to high in the majority of studies. Only one study explored the influence of age on AG levels, and stratification was not employed by any study; the risk of confounding affecting the review outcome is therefore high. Several studies did not report relevant effect measures, such as OR, AUC or mean difference or failed to provide confidence intervals, leading to exclusion from this review. Furthermore, the overall severity of illness in the study cohort was sometimes not quantified by means of an accepted disease severity score, such as the Acute Physiology and Chronic Health Evaluation II (APACHE II), Sequential Organ Failure Assessment (SOFA) score or Injury Severity Score (ISS) in trauma patients. No studies reported short-term mortality outcomes, which may have been more appropriate as naturally the prognosis in critical care patients is heavily influenced by clinical interventions undertaken during the inpatient stay. Shapiro and colleagues found that a single lactate level drawn on admission has good discriminatory power to predict 3-day mortality (AUC = 0.8) but poor discriminatory power to predict 28-day mortality (AUC = 0.67) [36]. Lastly, few studies stated the types of intravenous fluids used and no study included the quantity of intravenous fluid used as a variable in multivariate analysis.

## Conclusion

The high degree of unexplained statistical heterogeneity, considerable diversity between patient cohorts and poor quality of primary studies, in particular the high risk of attrition bias and confounding, impact on the overall strength of evidence of this systematic review and meta-analysis. The majority of studies reported here do not support the use of the AG as a predictor of 31-day mortality, in-ICU mortality or in-hospital mortality. Therefore, based on the available evidence, the use of a single AG measurement for risk stratification in critically ill patients cannot be recommended. Further high quality research would be required to conclusively determine the validity of the AG as a predictor of mortality. However, the probable influence of intravenous fluids on AG levels and the substantial baseline variability between AG levels among healthy individuals may render the use of the AG problematic in clinical practice. In light of the growing body of evidence supporting the use of lactate concentration for monitoring of critically ill patients, it may be more worthwhile to focus efforts on increasing the capacity for lactate measurement in low resource settings.

## Additional files

Additional file 1: Figure S1. Forest plot of area under the ROC curves (AUCs) for corrected AG predicting mortality. Forest plot of a random effects meta-analysis of AUCs for the corrected AG predicting mortality; I^2^ = 67 %. In view of the high heterogeneity a pooled effect estimate is not shown. (PDF 9 kb)

Additional file 2: Figure S2. Forest plot of odds ratios (ORs) for corrected AG predicting mortality. Forest plot of a fixed effects meta-analysis of ORs derived by univariate logistic regression for the corrected AG predicting mortality; I^2^ = 5 %. In view of the high heterogeneity in meta-analyses of other effect measures a pooled effect estimate is not shown. (DOCX 605 kb)

Additional file 3: Figure S3. Forest plot of mean differences for corrected AG predicting mortality. Forest plot of mean differences in corrected AG between survivors and non-survivors; I^2^ = 0 %. In view of the high heterogeneity in meta-analyses of other effect measures a pooled effect estimate is not shown. (DOCX 491 kb)

Additional file 4: Figure S4. Funnel plot of mean differences. Funnel plot of the standard error of mean difference (SE(MD)) against the mean difference for observed AG. MD = mean difference; SE = standard error. (DOCX 46 kb)

Additional file 5: Figure S5. Funnel plot of area under the ROC curve (AUC). Funnel plot of the standard error of AUC (SE(AUC)) against the AUC for observed AG. SE = standard error. (DOCX 47 kb)

## Abbreviations

AG: Anion gap
APACHE II: Acute physiology and chronic health evaluation ii
AUC: Area under the ROC curve
AVPU: Alert, verbal, pain, unresponsive
CIs: Confidence intervals
DKA: Diabetic ketoacidosis
ENT: Ear nose and throat
GCS: Glasgow coma scale
ICCU: Intensive cardiac care unit
ICU: Intensive care unit
IQR: Interquartile range
ISS: Injury severity score
Mdn: Median
MI: Myocardial infarction
OR: Odds ratio
PCI: Percutaneous coronary intervention
PRISMA: Preferred reporting items for systematic reviews and meta-analyses
Pro: Prospective
QUIPS: Quality in prognostic studies
Retro: Retrospective
SAPS: Simplified acute physiology score
SD: Standard deviation
SE: Standard error
SOFA: Sequential organ failure assessment
STEMI: ST-elevation myocardial infarction
