# Supplementary figures and images for: Anion gap as a prognostic tool for risk stratification in critically ill patients – a systematic review and meta-analysis
Source: BMC Anesthesiol. 2016 Aug 30;16:68. doi: 10.1186/s12871-016-0241-y (PMC5006450; doi:10.1186/s12871-016-0241-y)

Summary meta-analysis plot [random effects]

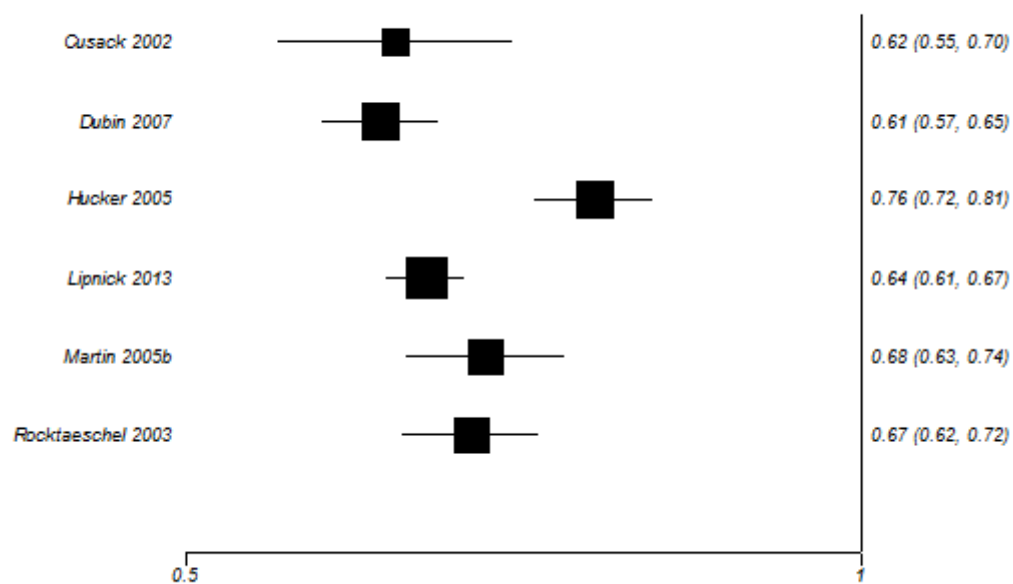

Supplement: Supplementary file 1 — Forest plot of area under the ROC curves (AUCs) for corrected AG predicting mortality. Forest plot of a random effects meta-analysis of AUCs for the corrected AG predicting mortality; I2 = 67 %. In view of the high heterogeneity a pooled effect estimate is not shown. (PDF 9 kb) [file 12871_2016_241_MOESM1_ESM.pdf]

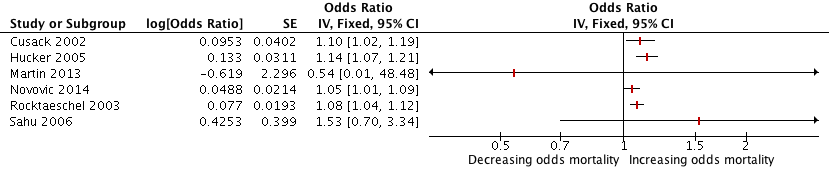


Supplementary material Figure 2

Supplement: Supplementary file 2 — Forest plot of odds ratios (ORs) for corrected AG predicting mortality. Forest plot of a fixed effects meta-analysis of ORs derived by univariate logistic regression for the corrected AG predicting mortality; I2 = 5 %. In view of the high heterogeneity in meta-analyses of other effect measures a pooled effect estimate is not shown. (DOCX 605 kb) [file 12871_2016_241_MOESM2_ESM.docx]

*
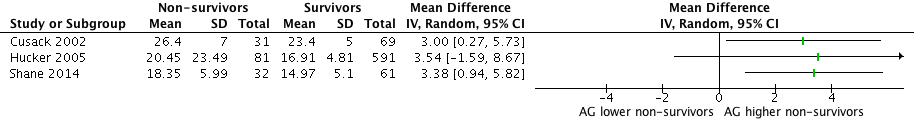
*

Supplementary material Figure 3

Supplement: Supplementary file 3 — Forest plot of mean differences for corrected AG predicting mortality. Forest plot of mean differences in corrected AG between survivors and non-survivors; I2 = 0 %. In view of the high heterogeneity in meta-analyses of other effect measures a pooled effect estimate is not shown. (DOCX 491 kb) [file 12871_2016_241_MOESM3_ESM.docx]

*
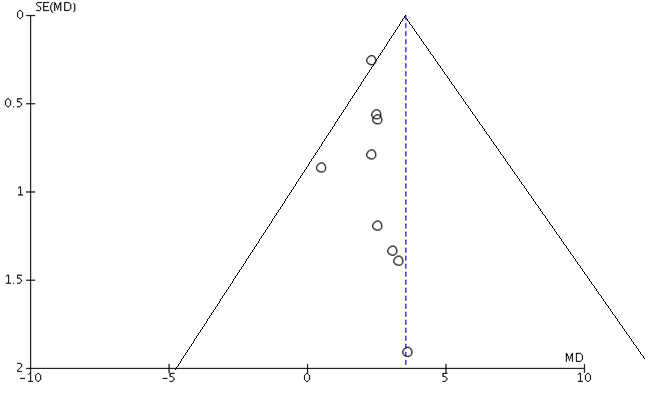
*

Supplementary material Figure 4

Supplement: Supplementary file 4 — Funnel plot of mean differences. Funnel plot of the standard error of mean difference (SE(MD)) against the mean difference for observed AG. MD = mean difference; SE = standard error. (DOCX 46 kb) [file 12871_2016_241_MOESM4_ESM.docx]

*
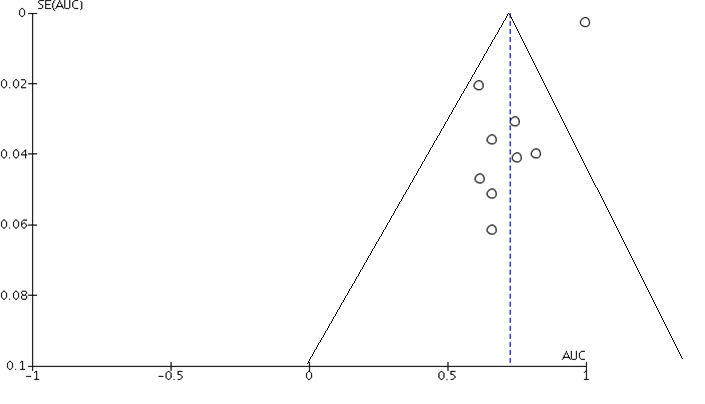
*

Supplementary material Figure 5

Supplement: Supplementary file 5 — Funnel plot of area under the ROC curve (AUC). Funnel plot of the standard error of AUC (SE(AUC)) against the AUC for observed AG. SE = standard error. (DOCX 47 kb) [file 12871_2016_241_MOESM5_ESM.docx]
